# Names and roles for the generalist doctor in Africa

**DOI:** 10.4102/phcfm.v2i1.242

**Published:** 2010-12-02

**Authors:** Steve Reid, Bob Mash, Joseph Thigiti, Lushiku Nkombua, Paul Bossyns, Ray Downing, Jan Heyrman

**Affiliations:** 1 Faculty of Health Sciences, University of Cape Town, South Africa; 2 Division of Family Medicine and Primary Care, Stellenbosch University, South Africa; 3 Moi University, Division of Family Medicine, Kenya; 4 Clinical Unit, Department of Family Medicine, Middelburg Hospital, South Africa; 5 Belgian Technical Cooperation, Belgium; 6 Department of Family Medicine, Moi University, Kenya; 7 Department of General Practice, Katholieke University, Belgium

## AN EMAIL DISCUSSION BETWEEN SIX FAMILY PHYSICIANS:

Dr Joseph Thigiti, Kenya

Dr Lushiku Nkombua, South Africa

Prof. Bob Mash, South Africa

Dr Paul Bossyns, Belgium

Dr Ray Downing, Kenya

Prof. Jan Heyrman, Belgium

## INTRODUCTION

The following dialogue between six family physicians was used as one of several discussion papers at the Regional Africa WONCA Conference in 2009 and was designed to stimulate debate and dialogue on the nature of Family Medicine in Sub-Saharan Africa. This is an edited version of an actual email exchange between March and June 2009, edited by the conference convenor, and reproduced with the kind permission of the authors.

**Dr Lushiku Nkombua:** The discipline of family medicine is growing actively in Africa, with the establishment of training departments for family medicine at some universities. South Africa (SA) is the torchbearer in this regard, having departments of family medicine in all the medical schools. The post-graduate training in family medicine in SA confers a Masters’ degree in family medicine at the end of 4 years of training. The accreditation authority in SA (Health Professions Council) registers the training in the specialist register. 

The specialist family physician is considered as the lead clinician in the district health system, in the primary health care facilities and district (level one) hospitals.

*My own observations of the tasks required from the South African specialist lead me to ask the question as to what really are the speciality’s responsibilities: family medicine (care for families) or district health care services (responsible for health care delivery in the district)*.

**Prof. Bob Mash:** In most sub-Saharan countries, first-contact primary care is the responsibility of nurses or clinical officers and not doctors or family physicians. Is this situation simply a consequence of limited financial and human resources and a second-rate health care system for the poor that will never deliver quality primary care? Or is this an appropriate and cost-effective approach to primary care in our setting where a doctor can be effectively substituted for and is not needed? Or are we saying that the combination of a family physician with a group of primary care nurses is capable of a high-quality primary care service? If the first assumption is true, then should we be aiming at increasing the number of family physicians in primary care to take over from nurses? Will we ever have enough doctors willing to do this in the current training model? If the second assumption is true, then are we mainly training family physicians to work in a district hospital setting? If the last assumption is true, then we need to have sufficient family physicians that are skilled in primary care, to support nurses in a team approach. This also implies much greater inter-professional understanding and collaboration, as well as mentoring skills.

In Europe, it takes three years to train an already qualified doctor to deliver high quality primary care. In South Africa we assume that we can train a nurse in 1 year to do the same. How can this be possible? Is the European doctor unnecessarily over-trained, or are South African nurses completely unprepared for their role as primary nurse practitioners? Is their role actually the same? Are we clear as to what we expect of our primary care nurses and when they need to refer on to a doctor? When we train doctors, we talk about sophisticated decision-making and relational skills, dealing with uncertainty and complexity. But when we train nurses we talk about algorithms to deal with the common and simple conditions seen in primary care. Which perspective is more appropriate? Are doctors clear as to how much one can expect of a primary care nurse? If quality primary care is so dependent on an effective collaboration between these two practitioners, are we helping them to form an effective partnership during their training periods and when they work side by side in the health services? A brief consideration would suggest that doctors and nurses are often different in terms of their gender, socio-economic background, language, race, values, norms and identity as professionals −a lot to overcome in forming effective, trusting and respectful relationships. Primary care nurses, in particular, have no real identity as practitioners who assess and treat patients − most still see themselves as nurses who are able to do primary care as one of their tasks.

Regardless of this, it seems self-evident that in SA, the family physician will need to work and be competent to provide services at the district hospital.

*Will the emphasis on hospital-based clinical skills during training erode the treasured principles of family medicine that have been defined on the basis of primary care? Can a family physician be an excellent generalist in primary care and in hospital care?*

Alternatively, will training programmes inappropriately hang on to principles derived from European and American primary care and fail to produce family physicians that can deliver what is needed in our context?

**Dr Paul Bossyns:** I cannot agree more with Dr Nkombua. Ten or 20 years ago, everybody would have been speaking indeed of district medical officers or district health physicians. “This term completely covers the type of doctors that SA” (and many other countries) needs for their decentralised services and for the health districts they want to install. Districts also exist officially in the UK or in the Netherlands. Because of the number of specialists available in these countries, at the so-called district level, cure and care is assured by specialist doctors and the administrative and financial management of the district system is given to hospital managers and other high-level administrative and management staff. The first line is manned by family doctors who deliver first-line health care.

In SA, one could say that nurses and clinical officers (I think, in some cases) are playing the role of the typical family medicine doctor. Because they are not fully qualified for this function, they work in delegation of the district health physician. Therefore, the district physician has to supervise the personnel at a primary level and has to master specificities of that level of care. Probably this aspect of their work has led to the opinion that these district physicians can be called family doctors.

The typical family doctor has other functions though, which cannot be provided by a doctor working at the district level where the proximity to the home and the family is lacking.

Typically, the tasks of a family doctor can be described as follows:

- Because of proximity and knowledge of the family and the social context of the patient, family physicians are in the position of making a socio-psychological diagnosis in addition to the physical aspects of disease. For the same reasons, they can contextualise certain solutions of the disease and in other words, can make more realistic proposals of conduct for the patients.
- Family physicians are the gatekeepers to consumerism in the sense that they are the only ones that can make a summary and keep an overview of the medical history of the patient and can protect the patient against overambitious specialists. They can help the patient decide, for instance, whether to be operated on or to live (temporarily) with the given condition.
- They are also the gatekeepers for the system's financing: they decide to refer or not and can save money for the system by treating the patients outside of the specialist care or hospital environment.
- They are the only physicians that can, due to their proximity again, deliver continuous care; and patients can consult their family physicians at any moment. Family physicians are therefore also the (family) crisis managers.

The problem with family physicians is the fact that one has to leave the somatic paradigm of bio-medicine and that one has to master other skills. In practice, this remains very difficult and many family physicians cannot get up to the quality required as described above. They become demotivated because they do not see the ‘serious pathology for which they are being trained’. They are also far too often discriminated against by the so-called specialist doctors, who forget that they know more and more about less and less. The most difficult discipline in medicine is, without any doubt, that of the family physician, who needs a very broad basis of knowledge and who has to work in uncertainty (with fewer diagnostic means), but also because they are taking into account the well-being of the patient as a complete person, that makes everything relative. Where the specialists are sure of their diagnoses and treatment schedules, the family doctors have to treat a patient when they are not yet sure what they are going to do ‘de facto’.

Anyway, we could go on for a long time: these concepts are largely described in the literature. In my experience in Africa, I can see that the lack of these first-line ‘family medicine skills’ means that about 10% of the diagnoses made at health care level are simply wrong because psychosomatic diseases and psychological problems are not recognised as such and are always treated with vitamins, anti-malarials and so on. The wrong answer pushes the patient towards becoming a chronic complainer and somatises the complaints to the level of a handicap: a huge cost for the quality of life of the patient. If one opens his eyes to these conditions, a completely different pattern of diseases and complaints can be unveiled; a different type of skill is needed at the first-line services. In other words, it is by recognising these other levels of diagnosis that the need for family medicine physicians is created, which is different from the very objective need of district health physicians in SA.

**Dr Ray Downing:** I have no doubt that where chronic physical, social and psychological diseases predominate, the ‘family medicine skills’ that Bossyns describes must be at the heart of the primary care providers’ approach – whether in sub-Saharan Africa or in Europe.

*But what, then, do we call post-graduate-trained district medical officers in rural Africa? Who decides what the essence of family medicine is?*

**Prof. Bob Mash:** In the Western Cape province of South Africa, we started off in 1997 with only six official Family Physicians (doctors who were qualified and in designated posts). Now in 2009, we have approximately 20 involved with the Stellenbosch training programme alone. The Comprehensive Service Plan for the province anticipates an ongoing expansion and I would foresee that eventually we will have several qualified family physicians at each health centre and district hospital. All permanent medical officers may eventually be family physicians. The role of the family physician, therefore, also shifts with time as they become more numerous. In the first phase, we have seen that they have been very involved in sub-district clinical governance and leadership, as part of the overall transformation of district services post-1994 in South Africa. However, as the numbers increase, the managerial and administrative load is more shared and they are already engaging with a greater clinical load at the primary care-level. It is widely acknowledged that they are trained to be clinicians and not district managers. One must therefore see that the role is an emergent property of the transforming health system and the number of family physicians available.

**Dr Lushiku Nkombua:** Family medicine in the developed world is, in the majority of the cases, an office-based discipline where the physician sees patients mainly for periodic health examinations, early detection of diseases and prevention of complications when diseases are already in existence. I had the opportunity to do a locum for 18 months in Canada in 2002−2003; the practice I was part of was not different from what is done in the local (SA) primary health care facilities or the general practice surgery. The practitioner is rightly called a family physician because one sees the entire family; starting with the parents, who would bring their own children and hopefully the parent’s own parents. Rarely would the family physicians in the developed world be responsible for patient care in the hospital, although they remain available as advisors to the treating hospital team.

The main role of the family physician in SA is to improve the quality of primary health care services within the district health system. Furthermore the role will be to develop clinical coherence or the integration of all the different programmes and services currently operating in the district health system. Apart from clinical skills, such integration requires a sound working knowledge of how the health system functions, excellent communication skills and the ability to establish effective referral patterns between different health facilities within the district health system.

In conclusion, such a multi-skilled physician cannot merely be called family physician with reference to the term as it is used in the developed world, taking into consideration the uniqueness and the complexity of the practice in the African context.

*I am of the opinion that the so called ‘family physician’ in Africa has, rightfully, to be called a **district health physician**.*

**Dr Paul Bossyns:** Where I tend to disagree, is persisting in calling them family doctors. What is wrong with referring to them in terms of what they actually do: district medical officers? I insist that using the term ‘family doctor’ in the case of ‘district medical doctor’ remains an aberration in terminology. It also pushes us towards a wrong working hypothesis. Of course, ANY medical doctor, including other specialists, should have knowledge of the psychosocial dimensions of disease and should develop skills which are germane to family medicine. But they will never become real specialists in that field because they are not practising in the optimal setting to get the necessary experience. Just like primary care workers do not become cardiac specialists by remaining in their health centre (although they will be meeting quite a lot of cardiac patients), the district medical officer and specialist doctors will always be handicapped in gaining the proper skills of the family doctor. It is indeed, therefore, that family medicine is also recognised more and more as a specific speciality, not something which is learnt spontaneously by simply seeing patients in any setting.

If we can agree with that, the actual restriction of the African primary health care setting becomes obvious. Indeed, as rightly mentioned, district medical officers are asked to supervise primary care nurses. And indeed, they can do so for many aspects of primary care (the typical bio-medical and general attitude aspects) but they do not have the correct background to supervise the other psychosocial aspects. In Europe, various specialists can supervise and/or provide additional training for general doctors at the first-line care level, but the typical family medicine aspects of care are continuously gained by training, mainly through peer review, just like cardiologists improve themselves through peer review continuous training as well. This, of course, does not exclude in any way the very fruitful interactions between different specialities, but family medicine should be regarded as one as well.

Now, because district medical doctors are not the best placed to effectively specialise in typical family medicine skills, they are in this respect not the best supervisors of those health care workers who should have these skills at the primary level, but because of their limited training, do not have them. And there we get into a vicious circle, which in my opinion cannot be broken as long as non-medical staff is in charge of the primary care. I dare to say that I have a very large degree of experience in supervision in very different African contexts, but I have never seen very significant improvements in the psychosocial diagnostic skills (besides basic attitudes and communication skills, of course, which all health workers should have). It means that there is a definite limit in the health care system which makes use of delegation of tasks because of a lack of human resources. It means, also, that in the (maybe very) long run, African countries will also need medical practitioners at the primary care level (actually, in urban settings everywhere in Africa, private practitioners are already doing it − though one can easily question the quality).

This is not an appeal to SA to say that their priority today should be different from training district medical officers. But let us not confuse terminology and identify family medicine with the typical skills and objectives of family medicine and keep typical district medical officers for the specific skills needed at that level of care. Also in South Africa, sooner or later, gynaecologists will say that they are more qualified for dealing with gynaecological problems at the district hospital and surgeons will claim they are better than district medical officers to perform appendectomies, because district medical officers have been delegated tasks which in other countries are done by specialists because they have the (human) resources to do so. This is not at all to say that I assume that, by definition, SA or any other country should necessarily evolve like the health care system in Europe and America.

*In summary, if we keep on confusing family medicine with district medical skills, the typical family medicine skills will be insufficiently developed and the weaknesses in the primary care setting will not be tackled in the proper way.*

**Prof. Bob Mash:** In South Africa, we are clear that the family physician and the district manager and/ or medical officer are two separate roles. In the Western Cape, each district and health facility has a full-time manager, who is separate from the family physician. The family physician is seen primarily as a clinician. Some are employed full time in primary care at large health centres and work in a team with other medical officers and nurses. They are the most highly trained clinicians in the team and tend to see the more complicated patients. They also provide in-service training and support to the team members. At a sub-district level, they are involved in clinical governance and are called upon by the district management to participate in the planning of health services.

Their training includes at least 1 year working full time in a primary care context. In our training programmes, we now train all family physicians to be able to work independently at the district hospital and primary care levels. After training, some will take posts at district hospitals and some at health centres in primary care. For many working in district hospitals, they must still work regularly at satellite primary care facilities in their sub-district.

**Dr Ray Downing:** A few thoughts on this rather interesting exchange: Dr Bossyns makes clear in his first contribution the specific attributes of family medicine and in his second, the plea to keep this set of skills and attributes separate from those of the district medical officer. I sense in these comments:

1. an affirmation of the roles of hospital-based generalist at the district level
2. a recognition that this person may not be the best to supervise the non-doctors (nurses, clinical officers) who are giving first-contact primary care and yet
3. an assumption that these specific ‘family medicine’ skills are as necessary in rural Africa as in Europe.

I quite agree with (1) and (2). I am less sure about (3). There two main reasons:

The family medicine roles Bossyns cites are vital when the epidemiology of a community is predominantly chronic disease together with a great deal of psychosomatic issues. Both, of course, exist in rural Africa, but in my experience (10 years working as a family doctor in North America, 20 years working in Africa), the epidemiology is quite different. The epidemiology of rural Africa is still dominated by acute infectious disease, trauma and obstetric problems. Yes, the epidemiologic transition has begun and is most visible in cities and big towns; perhaps it is there that family medicine skills are particularly needed. But those skills are less vital where chronic disease is still not common and where many people still use traditional methods (including family, community and religion) to deal with family crises.

Bossyns guesses 10% of health centre diagnoses are wrong because ‘family medicine skills’ are not employed to uncover psychological issues. I agree – but that means 90% of the time the current system might be adequate. My wife (a family doctor) thinks the misdiagnosis rate is much higher than 10% − not because psychosomatic issues are missed, but because meningitis is misdiagnosed as malaria or typhoid and so on. In other words, the deficiency is not of the ‘family medicine skills’ so important in Europe and the US, but the deficiency is in mastery of skills in the somatic paradigm – regardless of whether the provider is a nurse, clinical officer, or doctor.

*Because of this, we in Kenya have focused on training doctors for the district level. We call this family medicine because we are training for the first doctor-level care in our context, just as Europe and North America have family medicine as the first doctor-level care in those contexts.*

We are training generalists – and are aware that generalist skills here may not be the same as generalist skills in a different epidemiology, economy and health system. If family medicine means context-specific generalists or first doctor-level care for a given community, then we are training family doctors. But if family medicine means a field that specialises predominantly in knowing the social context, that is aware of psychological factors, committed to one doctor-one patient continuity, skilled in managing family crises, being a gatekeeper for consumerism and for the system’s financing – but does NOT train in emergency obstetric care, life-saving surgery, accurate somatic diagnosis and treatment of very sick people, then we are not training in family medicine.

*So a vital question arises: is family medicine the same worldwide, or is it truly context-specific? Is there a one-size-fits-all definition?*

**Dr Paul Bossyns:** I am sorry to rather disagree with some of the remarks. Ten per cent of misdiagnosis only on the basis of psychosomatic disease or frank psychiatry, which are not the only aspects, of course, where typical family medicine skills are needed (indeed, also for chronic diseases, for instance) is high and important. This is not suggesting at all that other misdiagnoses are not made and would not be important, which is a reason (one of them) why supervision by district medical officers remains crucial. The 10% is not a guess. Several studies, conducted by psychiatrists and others, have very similar figures in different settings in Africa (and as a matter of fact, in the rest of the world as well). I agree fully that this means that 90% accurate care is very good (though we have also both modified this figure), which is the reason why I am seriously defending the primary care settings in Africa. Let us not forget that besides misdiagnosis because of neglect of the psychosocial aspects of disease, simple adherence to therapy, flight into inaccurate traditional medicine and late diagnosis and rejection by families, are also aspects of lack of psychosocial diagnosis and care.

To summarise my thoughts, I would disagree with the fact that family medicine skills would be less needed in Africa than elsewhere, because of a different epidemiology. We detect it, maybe, less, because we are biased/occupied by the sometimes severe (and unnecessary) physical suffering of people in Africa in resource-poor settings, but my experience with running psychiatric units at a district level, as well as psychosocial diagnosis in Africa, have convinced me largely of the high need for taking into account these aspects of care properly. I disagree that the somatic paradigm is always more important than the other and I would like to emphasise that many poor results in somatic medicine in Africa are because of a lack of skills in the psychosocial aspects of care.

**Dr Paul Bossyns:** Of course, family medicine is, just like any other aspect of medicine, context-specific and of course we need to train the doctors in those skills that are mostly needed within the context we are living in, but does that mean that we have to call district medical officers family doctors whilst they are not (or hardly) more occupied with families than any other specialist doctor? Why do we have to stick to the title of family medicine to cover the function of a district medical officer? Is it not because a certain type of doctor is a ‘first-level, doctor-care person’ that they should get the family doctor title? This is not simply a semantic question. If we call these doctors ‘family doctors’, we might actually get away from defining the specific, organisational characteristics of the ‘first-line care’ (which equates to mostly health centres). Therefore I am pleading, NOT to change your priorities, nor to challenge the definition of your health care system according to your context, but to call a spade a spade: district health care as the first referral level with generalists (not family doctors) and health centres as primary care centres, where aspects of family medicine are most important (amongst other things and yes, of course, also somatic diagnosis). If we do not do so, we might actually fall into the trap of health centres performing ‘supermarket’ medicine, where one person is responsible for taking blood pressure, another for measuring the belly, yet another for estimating haemoglobin levels and so on: a way of performing medicine which is, unfortunately, not just theory (observed in most African settings, including South Africa and more so, even in urban settings where they have plenty of health staff). If on the contrary, we do recognise (all) the specific function(s) of the primary care level, although we might not achieve all of them to the same degree at the same moment of development of the system, we actually might grow in all of them, instead of developing one aspect to the detriment of another.

I suggest that you call post-graduate-trained district medical officers just that, because that is what they are and you should be proud of it.

**Prof. Bob Mash:** This discussion also raises for me the question of how we can train primary care nurses to develop some of these more holistic skills. In Africa, should we not regard primary care nurses and clinical officers as potential practitioners within the discipline of family medicine? Currently, nursing training and culture encourages a task-orientated approach where comprehensive care is split up into a series of tasks that different people perform. This goes against the type of empathic and relational primary care that has been highlighted by Dr Bossyns. Should family medicine and primary care nursing not embrace each other more?

**Prof. Jan Heyrman:** What I’m going to state comes from my experience with an exercise we did in Europe; let’s be clear on that. So it is only a proposal for you all to judge if this is appropriate for Africa.

In the past In Europe, we had different ‘professional definitions’ which, for us, took too much into account the practical opportunities and the organisational context. We said that until now, we had defined the position of ‘family medicine’ from its ‘place in the health care system’. With all the changes that we have also had in Europe (e.g., all the new eastern countries) health care systems are too different and too unstable. We need a new definition that starts from the discipline itself.

And that is what we initiated in 2002, starting from the question: what makes this discipline so different from others, so specific? We defined, in many broad discussion forums, 12 characteristics as fundamental differences. And consequently, we have written down in the educational agenda, which core competencies should be taught in a training programme leading to this recognised discipline. In the Miller pyramid, we deliberately stopped at the level of competencies. I will not go into the performance aspect, because that is too related to the real practicalities, possibilities and opportunities of each concrete health care system.

The logic, then, is not to change, nor adapt the discipline in itself, but to reflect where in your health care system this type of ‘family medicine specialist’ is needed, where it is affordable and opportune. If you define its competencies clearly, it is also necessary to define the types of tasks that can be better, cheaper, or to an acceptable extent, be taken up by professionals with other competencies.

*Shouldn’t you, then, first agree that you should stick to the international definition of family medicine? And to what extent does this professional, the district medical officer, differ from other health care professionals whose skills are used very often (we know them in Europe, also)?*

I learned that the district medical officer comes mainly from a tradition of public health. But what does that mean in terms of specific competencies? Why not try to define it? Eventually you can add the other available competencies, like the primary care nurse and other health care workers.

In a second step, you can go to the different levels of care organisation that exist and you can define locally what the tasks are and what the needed competencies are. I have seen some good schematic overviews in your different publications.

In a third step, the teams need to be constructed, with the different complementary competencies that are needed at the chosen level, to respond to the demands at that level. It will need to deal with the different mixtures of professions and their competencies; ‘interdisciplinary teams with complementary competencies’ is the buzzword.

**Dr Ray Downing:** Interesting proposal. The question remains: who was involved in drafting ‘the international definition of family medicine’. Were there African family medicine academics in that process, reflecting the African situation?

**Dr Joseph Thigiti:** Training of doctors in Kenya is predominantly biomedical with two endpoints. After finishing undergraduate training, doctors either remain as medical officers or proceed at different times to study specialist post-graduate courses, which are often limited.

Subsequent scope of medical practice for medical officers is determined by their employer and the presence of other cadres of health providers working with them.

Medical officers are the majority cadre of doctors providing health services in Kenya.

Family medicine training in Kenya began in 2005. The district physician described in SA is equivalent to the District Medical Officer of Health (DMOH) in Kenya. This is a distinctly public health administrative position occupied by a medical doctor, preferably one who has done post-graduate training in public health. Their work is to supervise primary health care at the district, usually located either at the head of the district offices or at the district hospital.

The recent inception of family physician training in Kenya is conceived from the need to have a competent bio-psychosocial endpoint training programme for medical officers interested in being primary care providers. In a country with a high disease burden, the family physician is being trained to be competent in offering primary urgent, emergency medical, surgical services with problem-orientated care to individuals and to work with the DMOH and other health providers involved in primary care to support health prevention, promotion, maintenance and adjustments. Currently nurses, clinical officers, medical officers and the DMOH have little psychosocial training and how it relates to the bio-psychosocial morbidity and mortality burden. Amongst the five roles envisioned for the family physician in Kenya (health provider, leader, teacher, life-long learner and primary care researcher), teaching and providing leadership to other members in the health facility will be crucial for the satisfactory delivery of a holistic health care service to the community.

This suggests that the envisioned family physician in Kenya will have substantial clinical work to do. Unlike the DMOH, they will work from a health facility that is close to the community and that can offer primary care services as the most senior doctor trained in primary care. They are the doctors being trained to provide primary care to the father, mother and child (family) competently and therefore will be called a family physician.

*This suggests that the envisioned family physician in Kenya will have substantial clinical work to do. Unlike the DMOH, they will work from a health facility that is close to the community and that can offer primary care services as the most senior doctor trained in primary care. They are the doctors being trained to provide primary care to the father, mother and child (family) competently and therefore will be called a family physician.*

**Prof. Bob Mash:** In my mind, I see the definition of family medicine as a discipline that can be articulated at three different levels. The first level speaks to the values of the discipline and the way that we see the world – our paradigm. I would anticipate that if family medicine is a global discipline, we should be connected by shared meaning and values. At the second level is the way in which the paradigm is embodied in the local district health care system. Contextual differences in the burden of disease, levels of development, finances and so on, will determine the way in which the discipline of family medicine is expressed organisationally. It may also determine which types of practitioners are regarded as members of the discipline or as potential members. The third level relates to the specific competencies and skills required of the practitioners − the scope of practice. Differences between countries and regions will become greater as you move down the levels. All of the above is, of course, in a process of constant flux, in line with changes in shared meaning and contextual circumstances – it is a dynamic process and a definition only defines the discipline at one point in time and often, for one part of the world.

*The definition of ‘family medicine’ can be articulated at:*

- *first level – values and paradigm*
- *second level – implementation in local district health system and*
- *third level – scope of practice of practitioners practice of practitioners*

